# GM-CSF, Flt3-L and IL-4 affect viability and function of conventional dendritic cell types 1 and 2

**DOI:** 10.3389/fimmu.2022.1058963

**Published:** 2023-01-12

**Authors:** Seyed Mohammad Lellahi, Waqas Azeem, Yaping Hua, Benjamin Gabriel, Kristin Paulsen Rye, Håkon Reikvam, Karl-Henning Kalland

**Affiliations:** ^1^ Department of Clinical Science, University of Bergen, Bergen, Norway; ^2^ Centre for Cancer Biomarkers (CCBIO), Department of Clinical Science, University of Bergen, Bergen, Norway; ^3^ Department of Immunology and Transfusion Medicine, Helse Bergen, Bergen, Norway; ^4^ Department of Medicine, Haukeland University Hospital, Bergen, Norway; ^5^ Department of Microbiology, Haukeland University Hospital, Helse Bergen, Bergen, Norway

**Keywords:** myeloid dendritic cells, conventional dendritic cells, DC viability, DC apoptosis, DC death, cytokine

## Abstract

Conventional type 1 dendritic cells (cDC1) and conventional type 2 dendritic cells (cDC2) have attracted increasing attention as alternatives to monocyte-derived dendritic cells (moDCs) in cancer immunotherapy. Use of cDCs for therapy has been hindered by their low numbers in peripheral blood. In the present study, we found that extensive spontaneous apoptosis and cDC death in culture within 24hrs represent an additional challenge. Different media conditions that maintain cDC viability and function were investigated. CD141+ cDC1 and CD1c+ cDC2 were isolated from healthy blood donor buffy coats. Low viabilities were found with CellGenix DC, RPMI-1640, and X-VIVO 15 standard culture media and with several supplements at 24hrs and 48hrs. Among multiple factors it was found that GM-CSF improved both cDC1 and cDC2 viability, whereas Flt3-L and IL-4 only increased viability of cDC1 and cDC2, respectively. Combinations of these three cytokines improved viability of both cDCs further, both at 24hrs and 48hrs time points. Although these cytokines have been extensively investigated for their role in myeloid cell differentiation, and are also used clinically, their effects on mature cDCs remain incompletely known, in particular effects on pro-inflammatory or tolerogenic cDC features. HLA-DR, CD80, CD83, CD86, PD-L1 and PD-L2 cDC membrane expressions were relatively little affected by GM-CSF, IL-4 and Flt3-L cytokine supplements compared to the strong induction following Toll-like receptor (TLR) stimulation for 24hrs. With minor exceptions the three cytokines appeared to be permissive to the TLR-induced marker expression. Allogeneic mixed leukocyte reaction showed that the cytokines promoted T-cell proliferation and revealed a potential to boost both Th1 and Th2 polarizing cytokines. GM-CSF and Flt3-L and their combination improved the capability of cDC1 for dextran uptake, while in cDC2, dextran capture was improved by GM-CSF. The data suggest that GM-CSF, IL-4 and Flt3-L and combinations might be beneficial for DC viability and function *in vitro*. Limited viability of cDCs could be a confounding variable experimentally and in immunotherapy.

## Introduction

Dendritic cells (DCs) are professional antigen-presenting cells, which are present in tissues and blood (1). DCs bridge innate and adaptive immunity by collecting and processing antigens to prime naïve T-cells (2). Natural DCs in peripheral blood are traditionally divided mainly into three subsets, myeloid/conventional DC1 (cDC1) and DC2 (cDC2), and plasmacytoid DC (pDC) (2). Recently, high resolution techniques have spurred further subclassification (3). CD34+ haematopoietic precursors in the bone marrow give rise to monocyte-DC progenitors (MDP). MDP can differentiate into monocytes and committed DC progenitors (CDPs). Committed DC progenitors can develop into pDC and pre-cDC, and eventually, CD141+ cDC1 and CD1c+ cDC2 arise from pre-cDC (4). Monocyte-derived DCs (moDCs) are most commonly used in DC-based immunotherapy in clinical trials (5). MoDCs generally are differentiated by culturing monocytes in media containing granulocyte-macrophage colony stimulating factor (GM-CSF) and interleukin 4 (IL-4) (6, 7). As the frequency of natural DCs is around 1% of peripheral blood mononuclear cells (PBMCs), using moDCs for clinical immunotherapy seems convenient as it provides a high number of moDCs *in vitro (* 5). Whereas moDC-based immunotherapy has been beneficial for a minority of patients, with increased progression-free survival, most clinical trials have shown inconclusive or ineffective results (8–10). It is likely that the full potential of DC-based cancer immunotherapy has not yet been realized. It is unclear how well *in vitro*-generated moDCs match the ability of natural cDCs to engage the adaptive immune system in a systemic attack on cancer cells. For these reasons, there is currently increased interest in the natural myeloid DCs in peripheral blood as an alternative to moDCs in future DC-based cancer immunotherapy (5, 11).

Pattern recognition receptors (PRRs) on DCs recognize both microbe-specific molecular signatures called pathogen-associated molecular patterns (PAMPs) and self-derived molecules derived from damaged cells known as damage-associated molecules pattern (DAMPs). Activation of PRRs orchestrates innate and adaptive immune responses (12). Toll-Like Receptors (TLRs) are well characterized PRRs. The TLR family consists of 10 members (TLR1-TRL10), and they are expressed in different immune cells including DCs. TLR4 is located on the surface of DCs and recognizes bacterial lipopolysaccharide (LPS). Intracellular TLRs recognize nucleic acids from pathogens and damaged cells (13), for example, TLR3 detects RNA from damaged cells, viral double-stranded RNA, and small interfering RNAs and can be stimulated experimentally by polyinosinic-polycytidylic acid (poly I:C). TLR7 recognizes viral single-stranded RNA, and TLR8 detects viral and bacterial RNAs (12) and TLR7/8 can be stimulated experimentally by R848 (resiquimod). There is a difference between cDC1 and cDC2 in terms of TLR expression. Q-PCR analysis showed that blood cDC2 expresses all TLRs except for TLR9, whereas cDC1 does not express TLR4, TRL5, TLR7, and TLR9, but expresses TLR1, TLR2, TLR6, TLR8, and with particular high expression of TLR3 and TLR10 (14).

Phenotypical markers, viability and function of DCs are dependent on their local microenvironment (15–18). The secretion of cytokines has a role in immune system homeostatis (19), DC differentiation (4) and viability (20, 21). The low numbers of cDCs in peripheral blood is one main reason that the field for decades turned to moDCs differentiated from more plentiful peripheral blood monocytes. In the present work, we have found that spontaneous apoptosis and cDC death represent an additional challenge toward their immunotherapeutic use. It is our impression that problems with viability of isolated cDCs have been under-communicated in the published literature and could be a confounding variable in experimental *in vitro* studies and in next-generation immunotherapy. Anti-apoptotic treatment of DCs has been reported to activate T-cell immunity (22–24). Improved cDC lifespan and control of apoptosis could potentiate cancer immunotherapy. In the present study we have therefore examined conditions that may improve the viability of cDC1 and cDC2 in culture, and among many variables the addition of Flt3-L, GM-CSF, and IL-4 to the growth medium showed significant effects.

The Fms-like tyrosine kinase receptor 3 (Flt3) is normally expressed by hematopoietic stem cells (HSCs) (25) or progenitor cells (26). The Flt3 ligand (Flt3-L)/Flt3 axis controls cell survival, proliferation, and differentiation *via* different signaling pathways, including PI3K, RAS, and STAT5 (27).

GM-CSF has been utilized to generate mouse DC-like cells from blood or bone marrow since 1992 (7, 28). Later, this protocol was refined, and a mixture of GM-CSF and interleukin-4 (IL-4) was commonly used to study mouse DC biology (6, 29). A recent study showed that treatment of bone marrow cells with GM-CSF resulted in the generation of moDCs resembling macrophages, and cDC-like cells that differed from cDC1 and cDC2 (30). The Flt3-L and GM-CSF combination is being used for differentiation of CD34+ cells to CD141+ cells *in vitro (* 31).

## Materials and methods

### Dendritic cell isolation

Peripheral blood mononuclear cells (PBMCs) were isolated from healthy blood donor buffy coats using Lymphoprep (Stemcell Technologies; Cat. No: 07811) density gradient centrifugation. Informed consents were obtained from all donors, and samples were anonymized according to the approval by the Regional Ethical Committee (#64205).

CD141+ cDCs and CD1c+ cDCs were isolated from PBMCs in two steps. First, the Pan-DC Enrichment kit (Miltenyi Biotec; Cat. No. 130-100-777) was used to remove unwanted cells from PBMCs to enrich CD141+ and CD1c+ cDC populations according to the Miltenyi Biotec protocol. By using this kit, unwanted cells were kept in the LS column in the magnetic field and untouched enriched DCs were collected for sorting. Next enriched DCs were labelled with Fixable Viability Stain 575V (FVS575V), and fluorescently conjugated mouse anti-human antibody cocktail for CD1c, CD141, CD11c, CD3, CD14, CD16, CD19, CD20, and CD56 (**Table 1**). FcR blocking (Miltenyi; Cat. No. 130-059-901) was added to the antibody cocktail to block unspecific antibody binding to FcR receptors. To isolate cDCs, doublets, dead cells, and lineage cells were excluded in our gating strategy, and thereafter CD141+ and CD1c+ cells were gated from the CD11c+ cell population (**Supplementary Figure 1A**). Gated CD141+ cells and CD1c+ cells were seeded in 96-well plates and incubated at 37°C with 5% CO_2_. Cell sorting was performed on a BD FACSymphony™ S6 Cell Sorter (BD Bioscience USA). The different studied timepoints, type of assay, and time of treatments are shown in **Supplementary Figure 1C**).

**Table 1:** List of used antibodies for sorting and analyzing of Dendritic cell.

Anti-Human Antibody	Clone	Cat number	Final concentration	Company
Sorting panel				
Fixable Viability Stain 575V		565694	1:1000	BD
CD14-FITC	18D11	21620143X2	1:50	Immuno Tools
CD16-Alexa Flour 700	B73.1	360718	1:50	Biolegend
CD11c-BB700	S-HCL-3	746106	1:140	BD
CD141-PE	AD-14H12	130-113-318	1:140	Miltenyi
CD1c-Alexa Flour 647	L161	331510	1:140	Biolegend
CD3-Brilliant Violet 421	OKT3	317344	1:140	Biolegend
CD19-Brilliant Violet 421	SJ25C1	363018	1:140	Biolegend
CD20-Brilliant Violet 421	2H7	302330	1:70	Biolegend
CD56-Brilliant Violet 421	5.1H11	362552	1:70	Biolegend
Analysis Panel				
HLA-DR-Horizon 500	G46-6	561224	1:50	BD
CD80-Brilliant Violet 786	L307.4	564159	1:50	BD
CD83-(PE-CF594)	HB15e	562631	1:50	BD
CD86-Brilliant Violet 711	IT2.2	305440	1:80	Biolegend
PD-L1 (CD274)-(PE-Cyn7)	MIH1	255983-42	1:10	Invitrogen
PD-L2 (CD273)-(APC-Cyn7)	MIH18	345516	1:120	Biolegend
Annexin V-FITC		V13245	1:20	Invitrogen
Fixable Viability stain 575		565694	1:1000	BD

### Media and cell culture

All cells including CD141+ cDC and CD1c+ cDC were cultured in CellGenix GMP DC medium (CellGenix; Cat. No. 20801-0500) unless otherwise stated. CellGenix DC and other used media were supplemented with 100 U/ml penicillin and 100 µg/ml streptomycin (ThermoFisher Scientific; Cat. No. 15140122). Cells were kept at 37°C with 5% CO_2_. In all experiments, 8000-10000 CD141+ cells, and 30000 CD1c+ cells were seeded in 96-well F-bottom plates except where otherwise stated. Cells were cultured in either CellGenix DC, Gibco™ RPMI-1640 (Fisher Scientific; Cat.No; 11530586), or X-VIVO 15 (Lonza, BE02-060F) in presence or absence of supplementary cocktail including: Human AB-Serum (10%; Sigma-Aldrich; Cat.No. H3667), HEPES (10mM; Merck; Cat.No. H3537), 2-Mercaptoethanol (50uM; Merck; Cat.No. M3148), L-Glutamine (2mM; Sigma-Aldrich; Cat.No. G7513), Non-essential Amino Acid (1X; Sigma-Aldrich; Cat.No. M7145), and Sodium Pyruvate (1mM; ThermoFisher Scientific; Cat.No. 11360070). DCs were stimulated at the beginning of culturing with 10ng/ml of lipopolysaccharides (LPS; *In vivo*Gen; vac-3pelps), or 12.5 µg/ml of Low molecular weight Polyinosine-polycytidylic (Poly I:C; *In vivo*Gen; tlrl-picw) plus 12.5 µg/ml of High molecular weight Poly I:C (*In vivo*Gen; vac-pic), or 2.5 µg/ml of R848 (*In vivo*Gen; vac-r848) for 4hrs, 24hrs, and 48hrs. The synthetic double-stranded RNA-analog, Poly I:C, and the single-stranded RNA-analog, R848, are agonists for TRL3 and TLR7/8, respectively. For cytokine treatment cells were treated with GM-CSF (100 ng/ml), IL-4 (20 ng/ml), and Flt3-L (100 ng/ml) or their combinations. Cells were seeded in media containing cytokines right after sorting and incubated at 37°C for 24hrs and 48hrs.

### Flow cytometry analysis

Plates were incubated at 4°C for 10min, and cells were detached from the plate bottom by pipetting. The expression of surface markers, such as HLA-DR, CD80, CD83, CD86, PD-L1 and PD-L2 (**Table 1**), was analyzed 24-48hrs after treatments. To determine the viability of DCs, cells were stained with both Annexin V and Fixable Viability Stain 575V (FVS575V) in 1x Annexin V binding buffer. In this study, cells were divided into three populations: (i) live-cell (Annexin V-/FVS575V-); (ii) apoptotic cell (Annexin V+/FVS575V-); (iii) dead cell (Annexin V+/FVS575+ or Annexin V-/FVS575V+). Cells were stained at room temperature in the dark for 30 min, followed by washing with PBS containing 0.5% BSA (Miltenyi; Cat. No. 130-091-376) and 1x Annexin V binding buffer. In all graphs, the percentage of viable/apoptotic/dead cells were measured among cells recovered from seeded cells. Flow cytometric analysis was performed on BD LSRFortessa™ Cell Analyser (BD, Bioscience, USA) and data were analyzed by FlowJo (FlowJo, LLC, Ashland, OR, USA). The gating strategy and the workflow are shown in **Supplementary Figures 1B, C**.

### Dextran uptake assay

CD141+ and CD1c+ cells were seeded in cytokine-containing media in 96-well plates, then cells were incubated with 0.5 mg/ml of dextran conjugated with FITC (Sigma; Cat. No. FD40) for 90min at either 4°C or 37°C. After extensive washing, DCs which were positive for FITC-dextran were measured by flow cytometry. The dextran uptake upon each treatment was calculated by subtracting background uptake and data presented as fold change; *i.e.* the percentage of positive DCs for FITC-dextran in treated cells versus the percentage of positive DCs for FITC-dextran in control cells.

### Allogeneic mixed leukocyte reaction (MLR)

On day 0, sorted cDCs were seeded in 96-well plates at a density of 8000-10000 for CD141+ cells, and 30000 for CD1c+ cells in media with or without the addition of different cytokine mixtures. In addition, CD141+ cells were treated with 25 µg of Poly I:C (12.5 µg/ml LMW and 12.5 µg/ml HMW) and CD1c+ cells treated with 2.5 µg/ml of R848) for 24hrs which were termed as “TLR-stimulated” in contrast to “not stimulated” parallels without Poly I:C or R848. For responder cells, monocytes were depleted from allogeneic PBMCs on day 0, and cells were rested in RPMI-1640 (Fisher Scientific; Cat No. 12027559) supplemented with 10% FBS (Fisher Scientific; Cat No. 10706143) for 24hrs. Next day (24hrs), monocyte-depleted allogenic PMBCs were labelled with CSFE (ThermoFisher; Cat.NO. V12883; 5uM/ml). CFSE-stained cells were mixed with extensively washed DCs in ratio of 5:1. To provide an optimal culture for T-cells, 50 U/ml of IL-2 (Immunotools; Cat. No. 11340023) and 10 ng/ml IL-7 (Immunotools; Cat. No. 11340073) were added to the media at the beginning of the co-culture, and the cytokines were replenished every second day. To prepare positive control cells, CFSE-stained lymphocytes were treated with CD3/CD28-beads (Gibco, Waltham, MA, USA; Cat. No. 11161D) and for the negative control, only CFSE-stained cells were cultured in parallel. The proliferation of allogeneic T-cells was analyzed after eight days using BD LSRFortessa™ Cell Analyser. Culture supernatants were stored for Luminex analysis.

### Luminex performance assay

Culture supernatants from the MLR experiments were collected after eight days for Luminex performance assay using Human XL Cytokine Premixed Kit (R&D systems Biotechne brands, Cat. No. FCSTM18B) to measure the following cytokines: IFN Gamma (IFNγ), IL-5, IL-6, IL-10, IL-13, Tumor Necrosis Factor alfa (TNFα). The procedure was performed according to the provided protocol from the company and the cytokines were measured on a Bio-Plex 200 System (Biorad, Hercules, CA, USA). Briefly, 50 μl of supernatant from each treatment was diluted 1:2 in calibrator diluent RD6-65, and 50 μl of diluted samples were added to a transparent-flat-bottom 96-well plate. 50 μl of microparticle cocktail added to each sample and standard and incubated for 2hrs. After washing, 50 μl of diluted Biotin-Antibody cocktail was added to each well and incubated for 1hrs. Then, wells were washed, and 50 μl of streptavidin-PE was added to each sample and incubated for 30min. Finally, wells were washed for the last time, and 100 μl of wash buffer was added to each well and the plate was incubated for 2min before analysis. All the incubation steps were performed at room temperature on the shaker at 800rpm. All the steps of washing were performed while the plate was standing on top of a strong magnetic field. The concentration of cytokines was calculated from a standard curve.

### Statistics

The flow cytometry data were analyzed by FlowJo™ v10.8.1 software (BD Life Science). The quantitative data analysis were performed by GraphPad Prism version 9.2.0 macOS (GraphPad Software, San Diego, California USA). Statistical significance was determined using student t-test or two-way ANOVA. Statistical significance was defined as p ≤ 0.05. For all figures, significance values are shown in grade (Not significant (ns), p ≤ 0.05 (*), p ≤ 0.01 (**), p ≤ 0.001 (***), p ≤ 0.0001 (****). The error bars represent ± S.D. Each Graph is representative of at least three independent biological replicates.

## Results

To demonstrate the functional ability of cDCs, this study optimized the isolation of cDCs from buffy coats and investigated the viability and function of CD141+ and CD1c+ cells in different culture conditions.

### Viability of cDCs dropped within 24hrs

**Figure 1:**
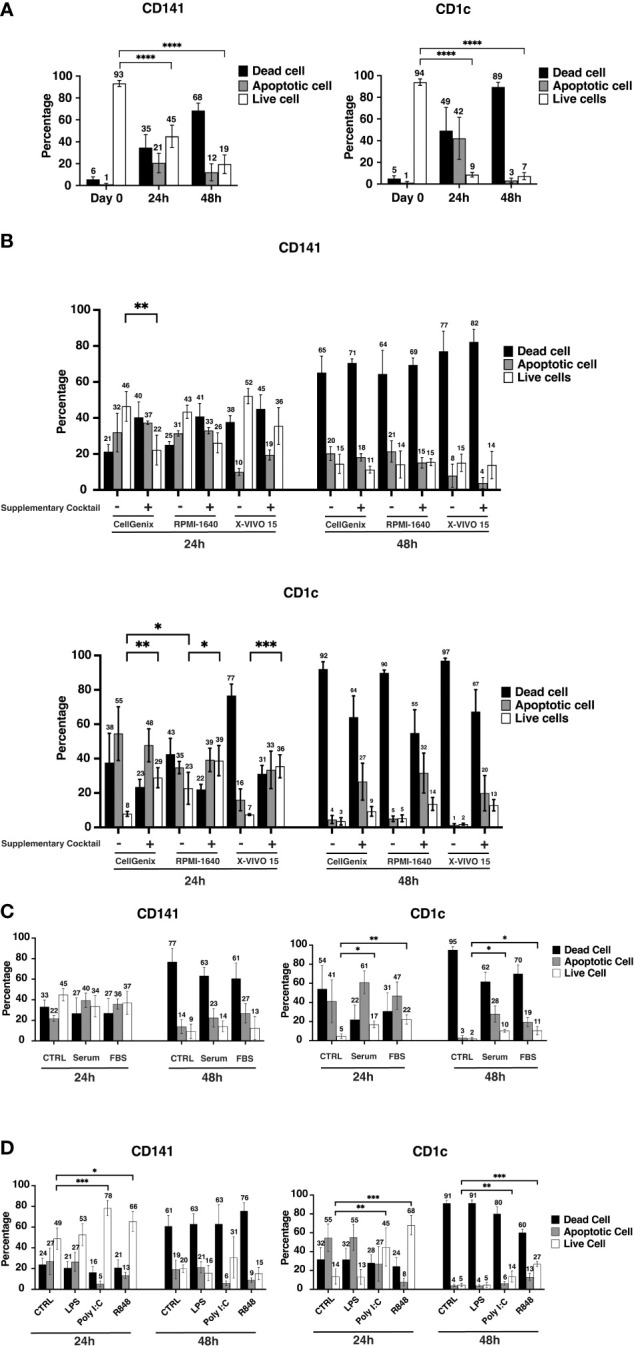
Conventional cell viability drops significantly within 24hrs. **(A)** After sorting, a portion of cells was stained with FITC Annexin V and Fixable Viability Stain 575V (FVS575V) and cells were analyzed by flow cytometry. Annexin V-/FVS575V- cells were considered as viable cells, Annexin V+/FVS575V- cells were considered as early apoptotic cells, and those cells which were Annexin V+/FVS575+ or only FVS575+ were considered as dead cells. Also, CD141+ cells (8000-10000 cells) and CD1c+ cells (30000 cells) were seeded in 96-well flat-bottom plates and cells were incubated at 37°C. The percentage of viable cells, early apoptotic cells, and dead cells were analyzed by flow cytometry after Sorting (Day 0), 24hrs (24h) and 48hrs (48h). The graphs are representative of four independent biological replicates. **(B)** 8000-10000 CD141+ and 30000 CD1c+ cells were seeded in 96-well plates either in CellGenix or RPMI 1640 or X-VIVO15 in the presence or absence of supplementary cocktail ((Human AB-Serum (10%), HEPES (1%), 2-ME (50uM), L-Glutamine (2mM), Non-essential a.a (1x), Sodium Pyruvate (1mM)) for 24-48hrs. The graphs are representative of three independent biological replicates. **(C)** CD141+ and CD1c+ cells were cultured in serum-free CellGenix medium or in the CellGenix medium containing FBS (10%) or Human AB-Serum (10%) for 24hrs and 48hrs. Data are extracted from three independent biological replicates **(D)** CD141+ and CD1c+ cells were treated with either LPS (10 ng), or Poly I:C (12.5 µg/ml of High molecular weight (HMW) Poly I:C plus 12.5 µg/ml of Low molecular weight (LMW) Poly I:C), or R848 (2.5 µg/ml), or left untreated for 24hrs, 48hrs. Cells were analyzed using flow cytometry to measure the percentage of viable cells, early apoptotic cells, and dead cells. Cells were analyzed after 24hrs to 48hrs. Bar graphs show percentages of viable cells (white), early apoptotic (gray) cells, and late apoptotic/dead (black) cells at different time points. The graphs are representative of four independent biological replicates and mean values ± S.D. are shown. *p* values were calculated using two-way ANOVA with ****, *p* ≤ 0.0001; ***, *p* ≤ 0.001; **, *p* ≤ 0.01; *, *p* ≤ 0.05.

To check the viability of human circulating cDCs, CD141+ and CD1c+ cells were sorted from healthy donor buffy coats and cultured in CellGenix DC media overnight. The viability of both subtypes was more than 90% shortly after sorting, 93% for CD141+ cDCs and 94% for CD1c+ cDCs (**Figure 1A**). After 24hrs, the viability of CD141+ cells dropped to 45%, while CD1c+ showed very poor survival, and the viable cells decreased to less than 10% (**Figure 1A**). The viability of CD141+ cells decreased even more, down to 19% after 48hrs culturing (**Figure 1A**). Furthermore, higher concentrations of cDCs or different types of plates did not improve viability (**Supplementary Figure 2**). The graphs represent the percentage of viable, apoptotic or dead cells amongst those cells that recovered after incubation in comparison to the percentage of viable/apoptotic/and dead cells of starting materials (**Figure 1A** and **Supplementary Figure 2**).

To investigate if the type of media and supplemented reagent have any effect on cDC viability, the three commonly used media for hematopoietic or DC culture, CellGenix DC, X-VIVO 15, and RPMI-1640 were compared in the presence or absence of following reagents, Human AB-Serum, 4-(2-hydroxyethyl)-1-piperazineethanesulfonic acid (HEPES), 2-Mercaptoethanol, L-glutamine, Non-essential Amino Acid, and Sodium Pyruvate (hereafter called “supplementary cocktail”). In the case of CD141+ cells at the 24hrs time point, cell viability was similar between the three tested media, and adding the cocktail to the media rather decreased viability, although the differences were not statistically significant between most of the tested conditions (**Figure 1B**). The viability of CD141+ cells after 48hrs did not improve in any of the culture media regardless of the supplementary cocktail (**Figure 1B**). CD1c+ cells were also cultured in similar media and supplementary cocktails for 24hrs and 48hrs. RPMI-1640 seemed better for CD1c+ cells and increased the viability (to 23%) in comparison to the other two culture media. In general, adding supplementary cocktails to media increased the viability of CD1c+ cells at the 24hrs but not at the 48hrs time point (**Figure 1B**).

Adding Human AB serum (10%) or FBS (10%) to cultural media did not help the viability of CD141+ cells either at the 24hrs or 48hrs time points. However, both Human AB serum and FBS improved the viability of CD1c+ in 24hrs and 48hrs time points (**Figure 1C**). It should be taken into account that although the viability of CD1c+ cells increased upon either Human AB serum or FBS, the percentage of viable cells was still low, approximately 20% at the 24hrs time point, and around 10% at the 48hrs time point (**Figure 1C**). As Human AB serum partially improved the viability of CD1c+ cells, therefore, the better viability of CD1c+ cells upon supplementary cocktail could be due to serum effect (**Figures 1B, C**). Moreover, adding 10% of albumin to CellGenix DC media did not improve the viability of CD141+ and CD1c+ cells (data not shown). Poly I:C and R848 enhanced the viability of both CD141+ and CD1c+ cells. The viability of CD141+ cells increased more using Poly I:C while R848 had a better effect on CD1c+ cells (**Figure 1D**).

### Cytokine treatment improved cDC viability

**Figure 2:**
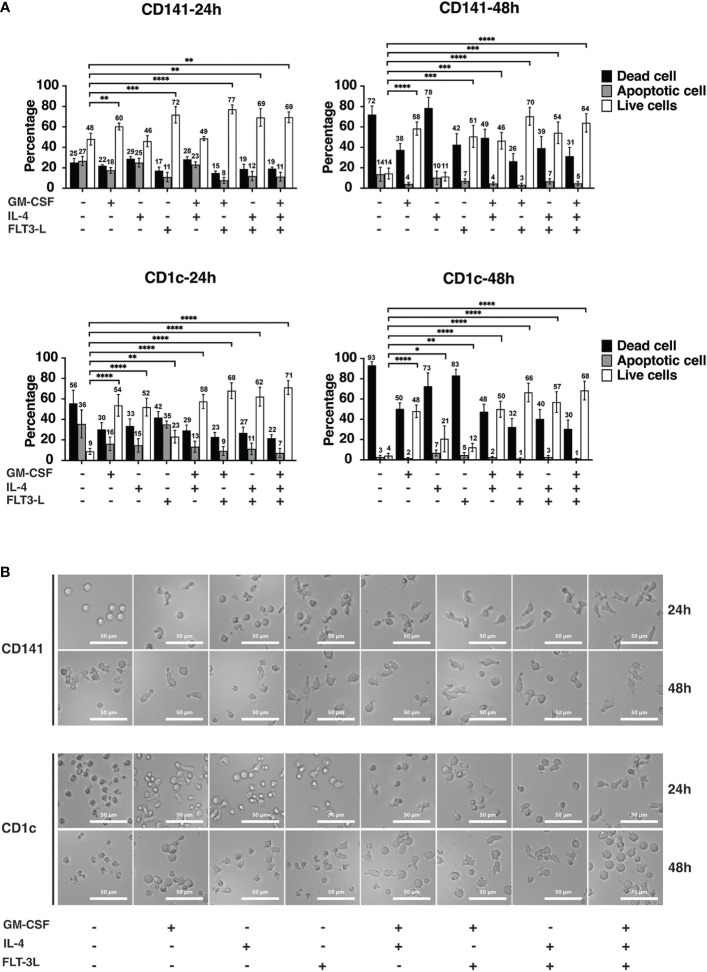
Cytokines improve the viability of dendritic cells *in vitro*. **(A)** CD141+ and CD1c+ cells were treated with different cytokines and cytokine combinations in Cellgenix DC medium as indicated. The concentrations were the following: GM-CSF (100 ng/ml), IL-4 (20 ng/ml), and Flt3-L (100 ng/ml). Cells were seeded in media containing cytokines right after sorting and incubated at 37°C for 24hrs and 48hrs. The graph indicates the summary of five biological replicates, and mean values S.D. are shown. *p* values were calculated using Student’s *t* test with ****, *p* ≤ 0.0001; ***, *p* ≤ 0.001; **, *p* ≤ 0.01; *, *p* ≤ 0.05. **(B)** For analysis of morphological changes upon cytokine treatment, cells were imaged after 24hrs and 48hrs using inverted phase-contrast microscopy. The experiments were performed at least three times, and representative images of one experiment are presented. The scale bar is 50 μM.

We checked if GM-CSF, IL-4, and Flt3-L or their different combinations could promote cDC viability *in vitro*. These data showed that GM-CSF and Flt3-L increased the viability of CD141+ cells at the 24hrs and 48hrs time points (**Figure 2A**). The combination of GM-CSF and Flt3-L had a synergistic effect on the viability of CD141+ cells at the 48hrs time point. CD141+ cells with Flt3-L alone showed 72% viability after 24hrs and suggested Flt3-L’s strong ability to increase viability (**Figure 2A**). CD1c+ cells were more responsive to cytokine treatment since treatment of cells with either GM-CSF, or IL-4, or Flt3-L promote cell viability (**Figure 2A**); Treatment of CD1c+ cells with a combination of GM-CSF + Flt3-L, IL-4 + Flt3-L, and GM-CSF + IL-4 + Flt3-L showed an additive effect in comparison with those cells which were treated with a single cytokine (**Figure 2A**). Interestingly, IL-4 did not affect the viability of CD141+ cells while it improved the viability of CD1c+ cells at both 24hrs and 48hrs time points. Flt3-L had a small but statistically significant effect on the viability of CD1c+ cells at both 24hrs and 48hrs time points (**Figure 2A**).

The functional and morphological characteristics of DCs have a direct effect on the phenotype of these cells (32). Phase contrast microscopy was used to investigate if media conditions change the morphology of CD141+ and CD1c+ cells. At the 24hrs time point, control cells for CD141+ and CD1c+ which were cultured in CellGenix DC media without any cytokine were round and spherical in shape. GM-CSF and Flt3-L treatment of CD141+ cells, resulted in cells with bigger size and longer pseudopodia and spikes (**Figure 2B**). The combination of cytokines further increased the effect on size, generation of pseudopodia structure and spikes in CD141+ cells (**Figure 2B**). CD1c+ cells were also affected by cytokine treatment after 24hrs resulting in morphology changes, and the combination of cytokine treatment increased size, pseudopodia structure, and spike formation. At the 48hrs time point, the same effect was observed, but it should be noted that most of the cells in the control (CTRL) group were dead, and also CD141+ cells treated with IL-4 and CD1c+ cells treated with IL-4 and Flt3-L had low viability (**Figure 2A**).

### CD141+ and CD1c+ immunophenotypes in different media conditions

**Figure 3:**
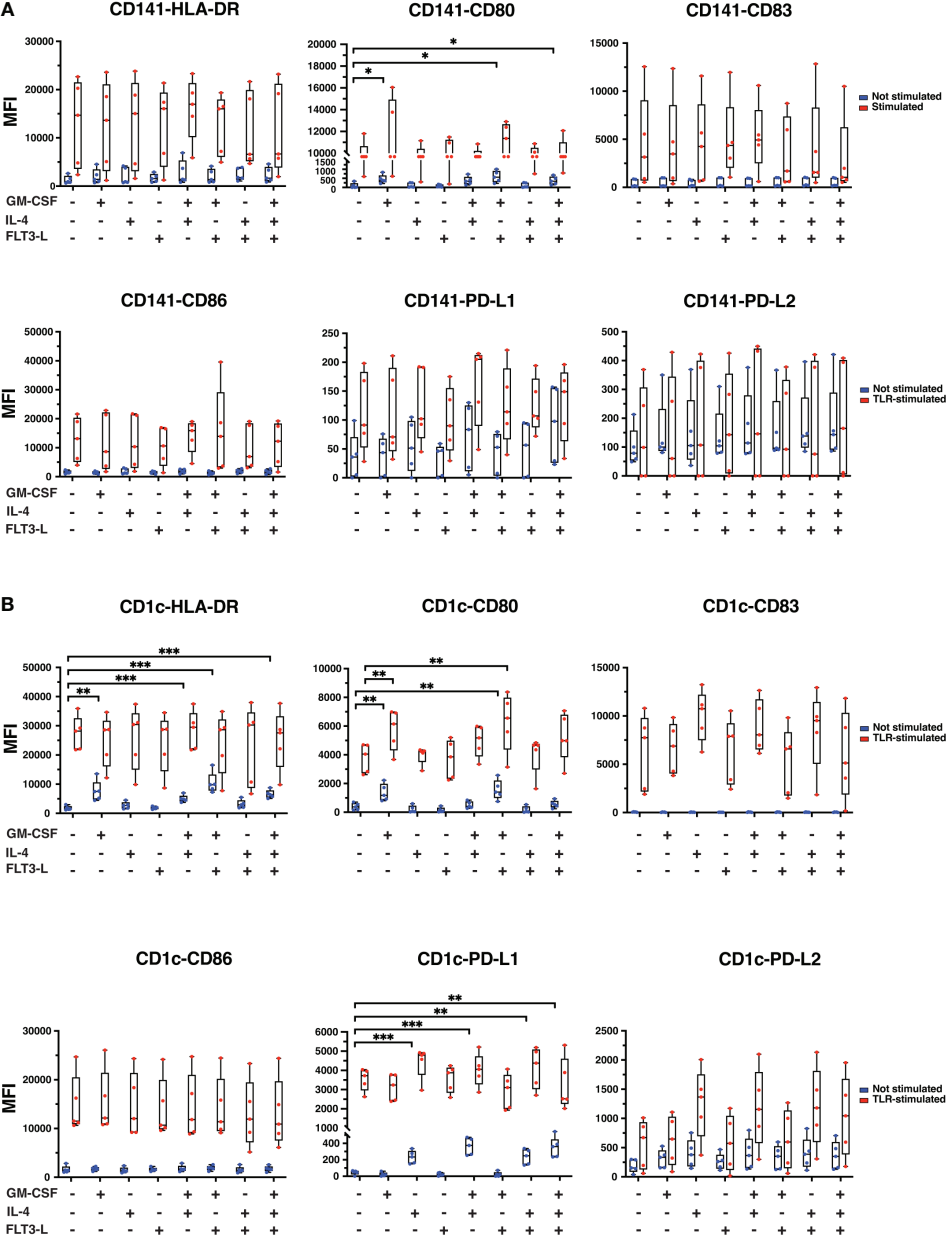
The expression of CD surface markers in CD141+ and CD1c+ cells upon different cytokine treatments in the presence or absence of TLR-stimuli for 24hrs. **(A)** CD141+ and **(B)** CD1c+ cells were cultured in media containing cytokines as indicated in the graphs. CD141+ cDC was treated with Poly I:C (12.5 µg/ml LMW and 12.5 µg/ml HMW) and CD1c+ cells were treated with 2.5 µg/ml of R848 or remained untreated (Not stimulated) for 24hrs. TLR-stimuli were added at the same time as cytokines. Surface marker expressions were analyzed by flow cytometry, and data are presented as mean fluorescence intensity (MFI). The data represent mean ± standard deviations (SD) of five independent biological replicates. Student’s *t* test analysis was used for calculation of *p* values ***, *p* ≤ 0.001; **, *p* ≤ 0.01; *, *p* ≤ 0.05.

IL-4, GM-CSF, and Flt3-L may affect DC membrane marker expression (33, 34). To check the effect of cytokine treatments on cDCs, sorted CD141+ and CD1c+ cells were cultured in different media conditions with or without TLR-stimulation. CD141+ cells were treated with Poly I:C and CD1c+ cells were treated with R848 to stimulate TLRs for 24hrs, and phenotypic markers, including maturation markers HLA-DR and CD83, activation markers CD80 and CD86 and inhibitory markers PD-L1 and PD-L2, were measured by flow cytometry after 24hrs. HLA-DR expression was strongly induced in both cDC types upon TLR-stimulation but addition of GM-CSF or IL-4 or Flt3-L did not cause further strong changes of expression of HLA-DR (**Figures 3A, B**). In CD1c+ cells, GM-CSF alone increased HLA-DR, and with significant further increase when R848 was added (**Figure 3B**). CD80 expression levels increased significantly in CD141+ cells with GM-CSF, GM-CSF+Flt3-L, and GM-CSF+IL-4+Flt3-L addition (**Figure 3A**), and with GM-CSF and GM-CSF+Flt3-L addition in both not stimulated and TLR-stimulated CD1c+ cells (**Figure 3B**). TLR stimulation increased the expression of CD83 and CD86, PD-L1 and PD-L2 in both cDC types, but combinations with either GM-CSF, IL-4 or Flt3-L neither increased nor reduced the expression of these surface markers much (**Figures 3A, B**). In cDC1+ cells, the basal expression level of PD-L1 increased significantly upon treatment with IL-4, GM-CSF+IL-4, IL-4+Flt3-L, and GM-CSF+IL-4+Flt3-L. Overall, isolated TLR-treatment induced the tested surface markers after 24hrs and this was in general not counteracted by the addition of individual or combined GM-CSF, IL-4 and Flt3-L (**Figures 3A, B**).

Additional experiments compared cDCs from Day 0 and Day 1 (24hrs) in medium without TLR-stimulation or addition of GM-CSF, IL-4 or Flt3-L (**Supplementary Figure 3**). In CD141+ cells, reduction of HLA-DR expression, and increasement of CD86 from Day 0 was observed; for other markers, there were no statistically significant differences between cells from the Day 0 and 24hrs time points (**Supplementary Figure 3A**). In CD1c+ cells, HLA-DR, CD80 and PD-L2 expression dropped after 24hrs (**Supplementary Figure 3B**).

CD141+ cells and CD1c+ cells were additionally treated with Poly I:C and R848, respectively for only 4hrs (**Supplementary Figures 4A, B**). TLR-stimulated cells expressed higher levels of CD80, CD83, and CD86 in comparison with non-stimulated cells while the differences between non-stimulated and TLR-stimulated samples were marginal for HLA-DR, PD-L1, and PD-L2, showing that co-stimulatory molecules have different response times following TLR-stimuli, suggesting that DCs might perform better functionally at selected time points after TLR-stimulation (**Supplementary Figure 4A**). In CD1c+ cells, surface markers expression remained unchanged in non-treated controls with the addition of TLR-stimuli (**Supplementary Figure 4B**). However, an increase was observed in HLA-DR, CD80, CD83 and CD86 expression in TLR-stimulated cells when treated with cytokines except for Flt3-L alone. PD-L1 expression in TLR-stimulated cells was also increased upon cytokine treatment except for GM-CSF, Flt3-L, and GM-CSF+Flt3-L treatment. PD-L2 expression was induced in TLR-stimulated cells only upon IL-4 treatment (**Supplementary Figure 4B**).

### GM-CSF and Flt3-L enhanced FITC-dextran uptake in cDCs

**Figure 4:**
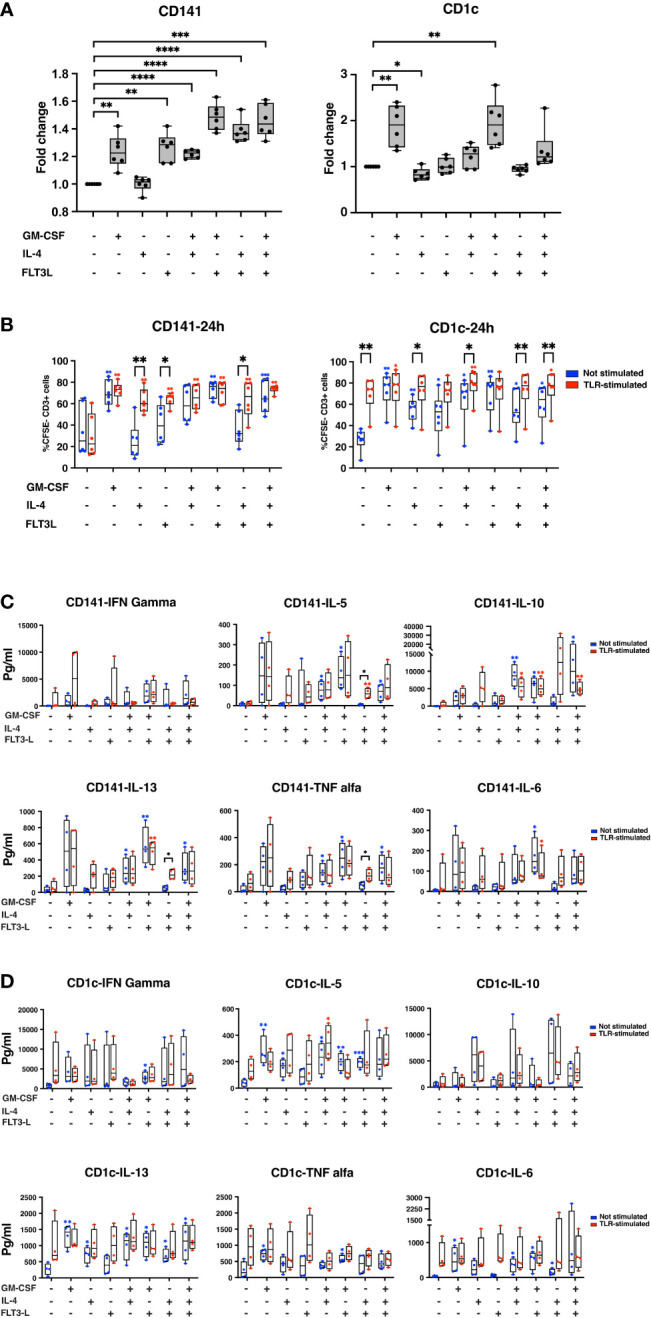
Cytokine treatment improves the performance of both CD141+ and CD1c+ cells. **(A)** CD141+ and CD1c+ cells were seeded in 96-well plates in different media conditions, as indicated in the graph. A day after, FITC-dextran was added to each media condition, and cells were kept at 37°C or 4°C for 90min. Those cells which were positive for FITC, are considered as cells that successfully uptake dextran-FITC. The percentage of positive cells for FITC was measured in each media condition, and the value from each condition was normalized with CTRL cells to calculate fold change. Graphs represent six biological replicates. The data is represented as fold change ± SD. Student’s *t* test analysis was used for calculation of *p* values ****, *p* < 0.0001; ***, *p* ≤ 0.001; **, *p* ≤ 0.01; *, *p* ≤ 0.05. **(B)** CD141+ and CD1c+ cells were sorted and cultured in different media conditions in the presence or absence of stimuli. After 24hrs, DCs were extensively washed and cocultured with CSFE stained monocyte-depleted PBMCs in the proportion of 1:5. “TLR-stimulated” denotes addition of Poly I:C to CD141+ cultures and R848 to CD1c+ cultures in contrast to “Not Stimulated” without these compounds. Cells were incubated at 37°C for eight days. IL-2 and IL-7 were added to the media every second day. Samples were analyzed using flow cytometry. Graphs indicate a summary of six biological replicates, and data represent the percentage of CSFE- CD3+ cells ± SD. Paired Student’s *t* test analysis was used for the calculation of *p-values* ***, *p* ≤ 0.001; **, *p* ≤ 0.01; *, *p* ≤ 0.05. **(C, D)** Media of MLR experiments were collected after eight days, and cytokine secretion was analyzed for each treatment as indicated. The data are shown as pg/ml, and paired student’s *t* test analysis was used for the calculation of *p* value ***, *p* ≤ 0.001; **, *p* ≤ 0.01; *, *p* ≤ 0.05. Graphs summarized results from four biological replicates. In **(B-D)**, the blue star showing summary of *p* values for not stimulated samples in comparison with control cells, and red star showing summary of *p* value for stimulated samples in comparison with stimulated-CTRL. The black stars show the summary of *p* values where not-stimulated and TLR-stimulated samples were compared in each cytokine treatment condition.

To activate T lymphocytes, DCs need to take up antigens from the environment and process them into peptides and present them in the context of MHC-I or MHC-II. DCs take up antigens in different ways, including macropinocytosis, receptor-mediated endocytosis, and phagocytosis (35). To check the ability of cells to take up antigens, CD141+ and CD1c+ cells were cultured in different media conditions for 24hrs as shown in **Figure 4A**, and then FITC-dextran was added to the media for 90min before analyzing cells with flow cytometry. Interestingly, the ability of CD141+ cells and CD1c+ cells to take up dextran was differently affected by cytokines. All media supplements except for IL-4 increased the ability of CD141+ cells to take up dextran (**Figure 4A**). Treated cells with GM-CSF+Flt3-L, IL-4+Flt3-L, and GM-CSF+IL-4+Flt3-L showed the highest ability to take up dextran in CD141+ cells (**Figure 4A**). In CD1c+ cells, only GM-CSF and GM-CSF+Flt3-L increased the ability to take up more dextran (**Figure 4A**). The cytokine treatment increased uptake of antigens almost two-fold in CD1c+ cells while the highest uptake of antigens in CD141+ cells were 1.4-fold. Moreover, the results showed that even Poly I:C-stimulated CD141+ cells, and R848-stimulated CD1c+ cells were able to capture dextran (data not shown) suggesting cDCs using a different strategy to take up new antigens (36, 37).

### IL-4 and Flt3-L treated cDCs induce proliferation of lymphocytes

The ability of DCs to induce T-cell proliferation is an important feature of the immune system to control both immune tolerance and immunity (38). We next analyzed the stimulatory activity of CD141+ and CD1c+ cells in an allogeneic mixed leukocyte reaction (MLR).

CD141+ cells stimulated by poly I:C did not increase T-cell proliferation significantly compared to control with medium only (**Figure 4B** left panel). Treatment of CD141+ cells with GM-CSF, however, increased T-cell proliferation both in the absence (not stimulated) and in the presence of poly I:C (stimulated). This contrasted with CD141+ cells treated with either IL-4 or Flt3-L where addition of poly I:C led to significant increase of T-cell proliferation (**Figure 4B** left). The combination treatments underscored that GM-CSF-treated CD141+ cells appeared not to be dependent upon additional poly I:C in order to achieve T-cell proliferation.

CD1c+ cells, in contrast to CD141+ cells, benefited from R848 TLR-stimulation, compared to control cells (**Figure 4B** right panel). Again, GM-CSF alone seemed to make also CD1c+ cells less dependent upon TLR-stimulation for their ability to increase T-cell proliferation (**Figure 4B** right). A similar pattern as for CD141+ cells was observed in combination treatments with both GM-CSF and IL-4 and GM-CSF and Flt3-L in that GM-CSF seemed to make T-cell proliferation less dependent upon additional stimulation with TLR-ligand (**Figure 4B** right). We performed a similar experiment where cDCs were treated with TLR stimuli for 4hrs, and then co-cultured with lymphocytes for eight days. According to this experiment, we did not observe any significant differences between the cytokine treatment group and control. No differences appeared between non-stimulated and TLR-stimulated CD141+ cells nor CD1c+ cells (**Supplementary Figure 5**).

### Cytokine treatments did not inhibit effect of TLR-agonists

Supernatants from the MLR were analyzed for cytokine secretion using the Luminex assay. IFNγ, IL-5, IL-6, IL-10, IL-13, and TNFα, were assessed (**Figures 4C, D**). A considerable variation was found for cDCs prepared from buffy coats of four different healthy donors, *e.g.* GM-CSF-treated plus TLR-stimulated CD141+ cells showed a pronounced IFNγ production in only 2 out of 4 MLRs (**Figure 4C**). Flt3-L and combination of Flt3-L and GM-CSF was associated with IFNγ production in subsets of CD141 cells (**Figure 4C**). TNFα was increased by GM-CSF in CD141+ cells both with and without TLR-stimulation (**Figure 4C**). Combinations between GM-CSF and IL-4 and GM-CSF+IL-4+Flt3-L appeared to have an inhibitory effect on IL-10 production in TLR-stimulated CD141+ cells. GM-CSF and combinations tended to increase both IL-5 and IL-13 both with and without Poly I:C treatment in CD141+ cells. Although a mixture of IL-4 and Flt3-L did not increase the basal level of IL-5 and IL-13 in CD141+ cells, this mixture significantly increased IL-5 and IL-13 secretion in TLR-stimulated CD141+ cells. IL-6 was induced in CD141+ cells only by a mixture of GM-CSF and Flt3-L. In CD1c+ cells, TLR-stimulation tended to increase all of IFNγ, IL-5, IL-6, IL-13, and TNFα, but not IL-10 (**Figure 4D**). GM-CSF tended to increase IFNγ and TNFα with or without R848. Both GM-CSF and IL-4 tended to increase IL-5 and IL-13 with or without R848. IL-4 and combinations tended to increase IL-10 with or without R848. Flt3-L stimulated TNFα together with R848 in CD1c+ cells.

## Discussion

Viability is one mandatory quality control of GMP-grade therapeutic DCs (39). In the present work we have found that although viability appears very high shortly after human cDC isolation, subsequent spontaneous apoptosis may be extensive following short-term culture. This problem may be a confounding variable both for *in vitro* experiments and for DC-based clinical trials, but it appears to be underreported in the published literature. DC lifespan *in vivo* has been most extensively investigated in mice models. Rapid death of isolated mouse DCs in culture has been found (40) and also of human Langerhans DCs in culture (41). In mice, the location of cDCs determines the lifespan of these cells (16–18). Published data revealed that the death rate of cultured mouse cDCs were more than 70% in 24hrs, and significantly higher than what was reported for the lifespan of cDCs in spleen (15). These data suggest that the microenvironment is critical for cDCs viability, and culturing cDCs *in vitro* induces stress signals resulting in spontaneous cell death in DCs (15). Bromodeoxy-uridine labelling has indicated a half-life of mouse spleen cDCs of approximately 48hrs (22). Parabiosis is a different approach to estimate DC lifespan in mice, with parabiont-derived cDCs in both spleen and lymph nodes disappearing within 10-14 days (17), and with even shorter and variable lifespans of different DC subsets in culture (21, 42). Presently, we have investigated culture conditions and lifespans of cDC1 and cDC2 isolated form healthy blood donor buffy coats. Viability was above 90% when the cells were examined immediately following their isolation. Upon culture for 24 and 48hrs the large majority of both cDC1 and cDC2 underwent spontaneous apoptosis and necrosis, and this was not remedied with different standard culture media, growth conditions or different serum concentrations.

RPMI-1640, X-VIVO 15, and CellGenix DC are commonly used media for DC culture (43) which in combination with supplementary reagents are expected to provide DCs with a proper culture environment (44). These supplementary reagents can balance the pH, and enrich media with serum, non-essential amino acids and sodium pyruvate, and finally remove free radicals (44). The supplementary cocktail has been used for culturing cDCs in some studies, while the effect of this cocktail on cDC viability has not been assessed (45–49). We demonstrated that the viability of CD141+ cells is similar in CellGenix, RPMI-1640, and X-VIVO 15. The additional cocktails did not improve viability in the current study. In fact, the viability of CD141+ cells rather decreased in media containing supplementary cocktails, suggesting that these reagents might be beneficial for moDCs but not for CD141+ cells. RPMI-1640 seemed to be the best basic medium for cDC1c+ cells and their viability improved by adding serum and FBS, and their viability increased even more in the presence of the supplementary cocktail.

TLR agonists play a critical role in the function of DCs (50, 51). In this work, we used Poly I:C and R848 agonists to stimulate TLRs of CD141+ and CD1c+ cells, respectively. Poly I:C has been shown to promote survival of mouse cDCs (15). According to one study, the viability of CD103+ cDCs in mice was improved upon antigen and TLR agonist stimulation, although viability dropped after 40hrs (45). However, another study reported that Poly I:C does not have any effect on cDC1 and cDC2 viability (48). In our present project, both poly I:C and R848 improved the viability of both cDC subtypes. Whereas it has been reported that LPS improved the viability of cDCs (15), we did not observe it in our experimental setting.

GM-CSF and Flt3-L have crucial roles in DC development and differentiation (52–54). Flt3-L is a growth factor that mediates proliferation, differentiation and development of cDCs *in vivo* and in bone marrow cell cultures with a reported relatively tolerogenic functionality (26, 55–58). *In vitro* studies have shown that GM-CSF has a key role in the differentiation of bone marrow-derived DCs, while IL-4 can support the development and maturation of DCs in the absence of GM-CSF (59, 60). There are several lines of evidence showing that GM-CSF enhances the viability of cDCs in mice (21, 61–63). IL-4 increases survival of cDC2 in atopic patients (20). In the present study, supplements of the cultures with GM-CSF, IL-4, Flt3-L, or certain combinations of these cytokines led to pronounced improvement of the survival of either cDC1 or cDC2 in comparison with multiple other culture conditions tested in parallel. The combination of GM-CSF and Flt3-L maintained the viability of these cells close to 70% after 48hrs. Similar positive effect of GM-CSF was observed on both CD141+ and CD1c+ cells, but the effect of Flt3-L on CD1c+ cell viability was marginal, while IL-4 was as effective as GM-CSF at the 24hrs time point. The combination of cytokines was beneficial for CD1c+ cells at both the 24hrs and 48hrs time points, indicating that cytokine combination is important for cDCs *in vitro*.

Similar to what we observed in this study *in vitro*, it has been shown that GM-CSF and Flt3-L synergistically maintain the total number of cDCs *in vivo (* 52, 64). It has been shown that GM-CSF might help the viability of DCs by inhibiting Bim-dependent apoptosis (65). Our data support that also IL-4 promotes viability of cDC2 in contrast to another report (20). According to cDC morphology and size, our results indicate that cDCs reached a semi-mature state in culture media containing cytokines. Expression of surface markers suggested that a more mature state could be achieved following addition of TLR agonists.

GM-CSF is commonly regarded as a potent pro-inflammatory cytokine (66). Still, seemingly conflicting results have been published in the literature when it comes to the ability of GM-CSF to support either pro-inflammatory or tolerogenic DC functions. GM-CSF has been used for the differentiation of mouse bone marrow cells to become tolerogenic DCs (67) and for *in vivo* treatment of both cancer and autoimmune conditions (67). From mouse bone marrow cells GM-CSF induces tolerogenic DCs with the ability to induce Tregs and ameliorate autoimmunity and degeneration in animal models (67). The complexity of GM-CSF, therefore, remains to be unravelled. The possible explanation is that the effect of GM-CSF is dose- and time- and context-dependent with signaling through several pathways, including JAK/STAT, MAPK, PI3K and canonical NFkB (58). The GM-CSF signal strength concept integrates both GM-CSF and its receptor abundances and associated regulatory circuits to understand effects on myeloid differentiation and functional outcomes of GM-CSF as a master regulator of myeloid cells and the T-cell-phagocyte interface (66, 68, 69).

Due to the versatile effects of GM-CSF on DC functions as published in the literature, we wanted to characterize functional outcomes and potential pro-inflammatory and tolerogenic features of GM-CSF of the cDCs isolated in our current study. In the absence of concomitant TLR-stimulation, GM-CSF for 24hrs did not affect the tested maturation, activation and inhibitory markers strongly in either CD141+ or CD1c+ cells. In general, the TLR-induced increase of maturation and expression markers was not counteracted by concomitant GM-CSF in the culture. Neither was TLR-mediated increase of inhibitory markers PD-L1 or PD-L2 counteracted by GM-CSF. The activation marker CD80 was, however, found to be markedly upregulated in TLR-stimulated CD141+ and CD1c+ cells following GM-CSF co-treatment for 24hrs. The conclusion for the tested concentration is that GM-CSF strongly improved viability of cDC1 and cDC2, but exhibited mostly a permissive role regarding the expression of the tested surface membrane markers. On the other hand, GM-CSF demonstrated its potential to promote several functional and pro-inflammatory features of both CD141c+ and CD1c+ cells, including FITC-dextran uptake and secretion of the signature Th1 pro-inflammatory cytokines IFNγ and TNF*a* in the MLR, although interperson variability was observed as found for other DC features previously (34). Caution should be taken in the interpretation of cytokine secretion changes in MLR supernatants, including the increase in Th2-polarizing cytokines IL-5 and IL-13 in several culture conditions. Differences could be secondary to increased viability of cDCs and thereby increased T-cell proliferation, irrespective of any polarizing ability of GM-CSF. The same would apply to the effects of Flt3-L and IL-4 in these assays.

It has been shown that CD141+ and CD1c+ cells have equal ability to dextran uptake (47). In CD141+ cells, all of the cytokine treatments except IL-4, increased the ability of these cells to take up dextran, and in CD1c+ cells only GM-CSF or mixtures of cytokines containing GM-CSF induced antigen uptake. The efficient antigen uptake is dependent on GM-CSF in chronic lung inflammation disease (70). *In vivo* study on mice in chronic lung inflammation disease showed that GM-CSF orchestrates antigen uptake, transport, and Th2 and Th17 polarization in lung DCs (70). Another study reported that Flt3-L is essential for robust immunity to subcutaneous immunization, and Flt3-L increases cDCs number and antigen uptake capability of migratory DCs and lymph node-resident cDCs (71).

In spite of Flt3-L’s essential role in myeloid cell differentiation and proliferation, the understanding of its role on individual DC subsets and its effect on mature DCs has been limited (33, 58, 72). Recently, protocols have been published with included Flt3-L for the preferential differentiation of either cDC2 (72) or cDC1 (31) from mouse or human precursors, respectively. Flt3 expression is preserved in terminally differentiated cDCs and pDCs, with possible pro-survival signaling through PIK3/AKT, but the effect of Flt3 signaling on mature DCs is still incompletely understood (33). One main conclusion of the present study is that Flt3-L addition to the basal culture medium significantly increased viability of both cDC types at the 24hrs and 48hrs time points, and this was potentiated by combinations of GM-CSF and IL-4. Similar to GM-CSF and IL-4, the addition of Flt3-L was permissive for the TLR-induced expression of tested surface markers after 24hrs. Additional expression of CD80 in both cDC types represented one exception. Flt3-L furthermore increased FITC-dextran uptake at 24hrs compared to control, increased T-cell proliferation in the MLR of CD141+ with concomitant TLR-stimulation. One important final conclusion is that it is important to be aware that cDC viability could be an important confounding variable in functional assays and immunotherapy using cDCs.

## Data availability statement

The original contributions presented in the study are included in the article/**Supplementary Material**. Further inquiries can be directed to the corresponding authors.

## Ethics statement

The studies involving human participants were reviewed and approved by Regional Committees for Medical and Health Research Ethics, Helse Vest. The patients/participants provided their written informed consent to participate in this study.

## Author contributions

Study conception and design: SL, K-HK, and WA; Experimental work and data collection: SL, WA, and KP; Analysis and interpretation of results: SL, K-HK, WA, HR, KP, BG and YH; Draft manuscript preparation: SL and K-HK. All authors contributed to the article and approved the submitted version.
