# Supplementary material for: GM-CSF, Flt3-L and IL-4 affect viability and function of conventional dendritic cell types 1 and 2
Source: Front Immunol. 2023 Jan 12;13:1058963. doi: 10.3389/fimmu.2022.1058963 (PMC9880532; doi:10.3389/fimmu.2022.1058963)
Supplement: Supplementary file 1 [file DataSheet_1.pdf]

A

Gating strategy for sorting

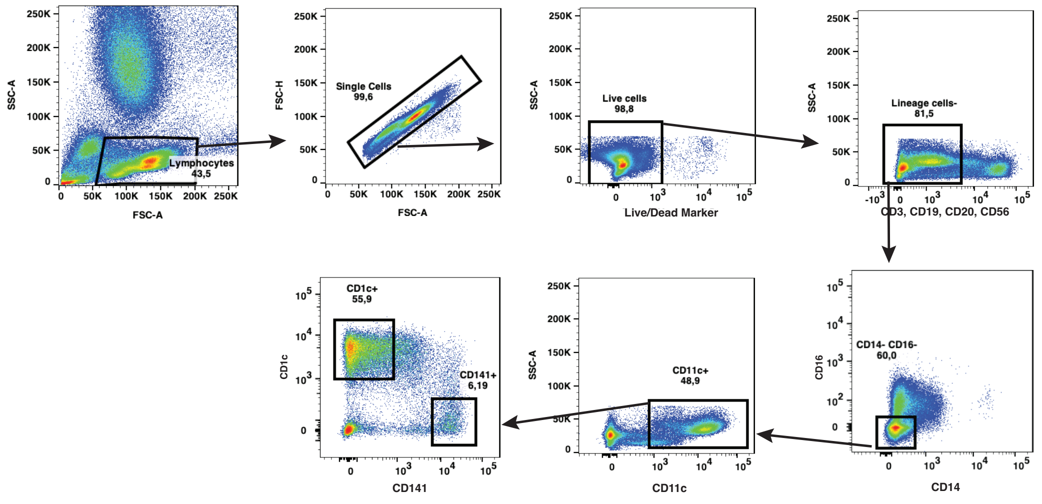

B

Gating strategy for cell analysis

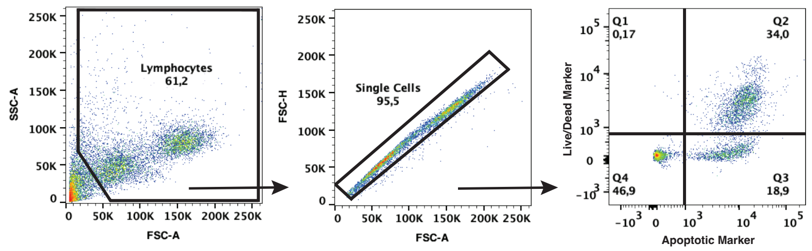

C

**Day 0**	1. PMBCs were isolated from one night old (16-18 hours) buffy coat (40 min)<br>2. Miltenyi kit Pan-DC enrichment of cDCs (20-30min)<br>3. Staining enriched DCs (30min) and CD141+ and cDC1c+ cell sorting (30min-60min)<br>4. Seeding cells and treatment of cells
**Day 1/2 (24h/48h)**	1. Imaging cells for morphological analysis (24hrs and 48hrs)<br>2. Collecting cells for viability and henotypic marker expression analysis (24hrs and 48hrs)<br>3. Ag uptake assay (24hrs)<br>4. Co-culture of DCs and lymphocyte for MLR assay (24hrs)
**Day 8**	1. Collecting MLR supernatant for Luminex analysis<br>2. Collecting cells from MLR for T-cell proliferation analysis

D

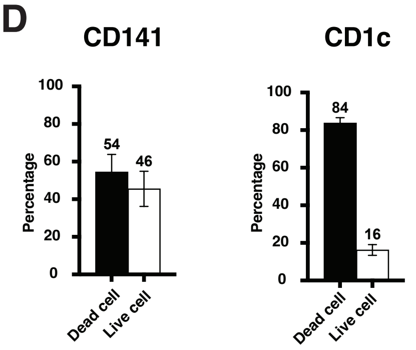

E

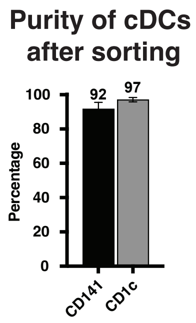

Supplementary Figure 1. Gating strategy for purification of CD141+ and CD1c+ cDCs. ***A.*** In the first step, doublet and dead cells were excluded from cDCs population. Then, lineage-positive cells (CD3, CD14, CD16, CD19, CD20, and CD56) were gated out, and CD11c+ cells were gated from lineage-negative. Conventional dendritic cells were gated and sorted from two populations based on the expression of CD141 and CD1c surface markers. ***B.*** The figure shows the gating strategy for analyzed cells. Only singlet live cells were gated for further analysis ***C.*** The table shows the definition of each time point and also explains the order of procedures, treatment and assay in this study. ***D***. CD141+ and CD1c+ cDCs were isolated using the CD141 (BDCA-3) MicroBead kit (Miltenyi Biotec; Cat NO. 130-090-512) and CD1c (BDCA-1) Dendritic cell isolation kit (Miltenyi Biotec; Cat NO. 130-119-475) thus minimizing physical pressure during the isolation step. Cell viability was analyzed after 24hrs. This data showed that the viability of CD141+ and CD1c+ cells dropped after 24hrs suggesting that other variables than physical pressure during sorting represent the main reason for the low viability of cDCs. ***E.*** The purity of CD141+ and CD1c+ cells were investigated after sorting, showing that the gating strategy is efficient for the isolation of high purity CD141+ and CD1c+ cDCs.
