# Supplementary material for: GM-CSF, Flt3-L and IL-4 affect viability and function of conventional dendritic cell types 1 and 2
Source: Front Immunol. 2023 Jan 12;13:1058963. doi: 10.3389/fimmu.2022.1058963 (PMC9880532; doi:10.3389/fimmu.2022.1058963)
Supplement: Supplementary file 2 [file DataSheet_2.pdf]

Supplementary Figure 2

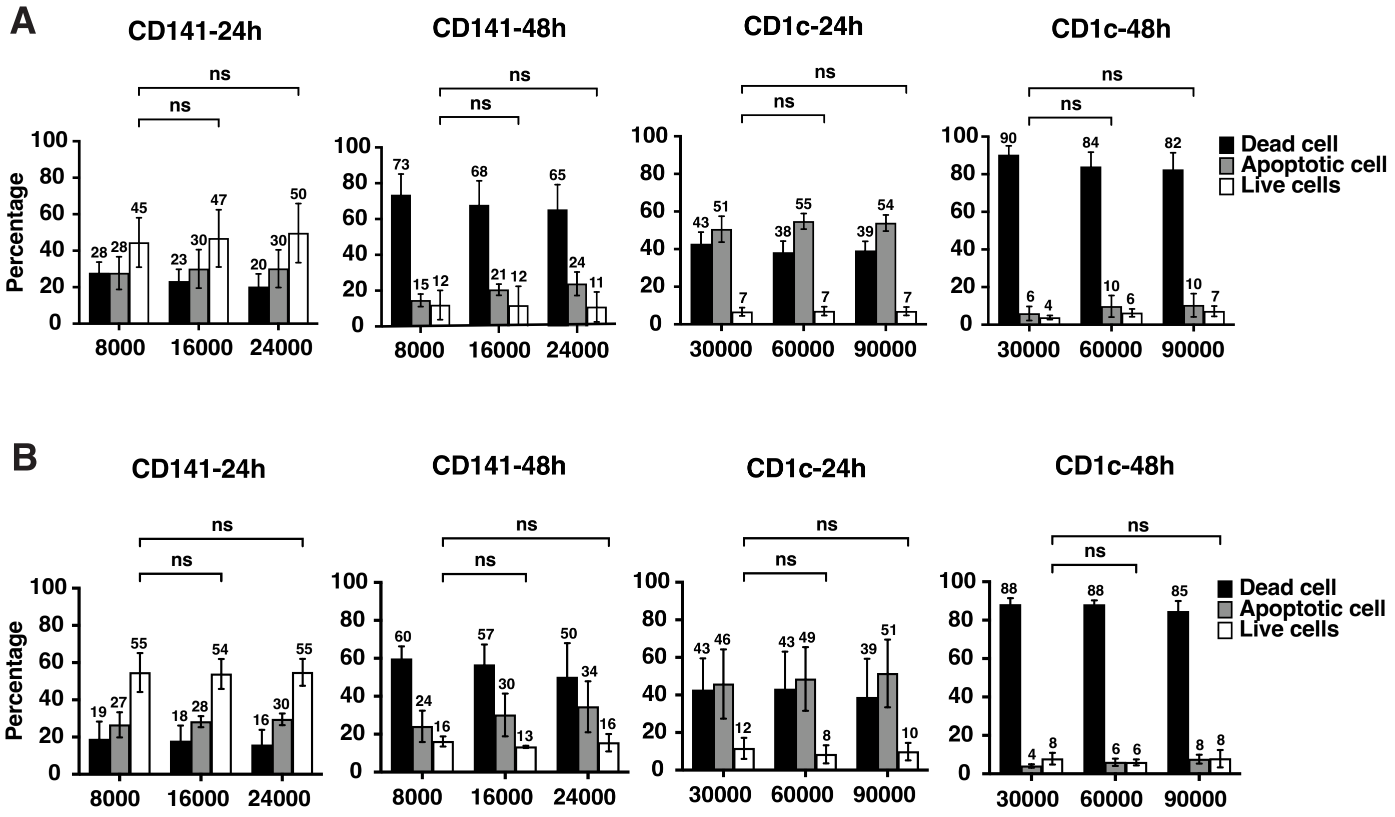

Supplementary Figure 2. The viability of conventional dendritic cells is not dependent on cell numbers and proximity in vitro. ***A.*** CD141+ and CD1c+ cells were seeded at different densities in a 96-well Flat-Bottom plate. The number of cells increased two times and three times per well, therefore, 8000, 16000, and 24000 CD141+ cells for CD141+ dendritic cells, and 30000, 60000, and 90000 cells for CD1c+ cells were seeded in a F-Bottom 96-well plate. Seeded cells were analyzed by flow cytometry after 24hrs and 48hrs. ***B.*** CD141+ and CD1c+ dendritic cells were seeded in a U-bottom 96-well plate. The cell number was doubled and tripled as explained in section A, and the percentage of the viable cells, apoptotic cells, and dead cells were analyzed after 24hrs and 48hrs. The graphs represent three independent biological replicates (ns, not significant).
