# Supplementary material for: GM-CSF, Flt3-L and IL-4 affect viability and function of conventional dendritic cell types 1 and 2
Source: Front Immunol. 2023 Jan 12;13:1058963. doi: 10.3389/fimmu.2022.1058963 (PMC9880532; doi:10.3389/fimmu.2022.1058963)
Supplement: Supplementary file 3 [file DataSheet_3.pdf]

Supplementary Figure 3

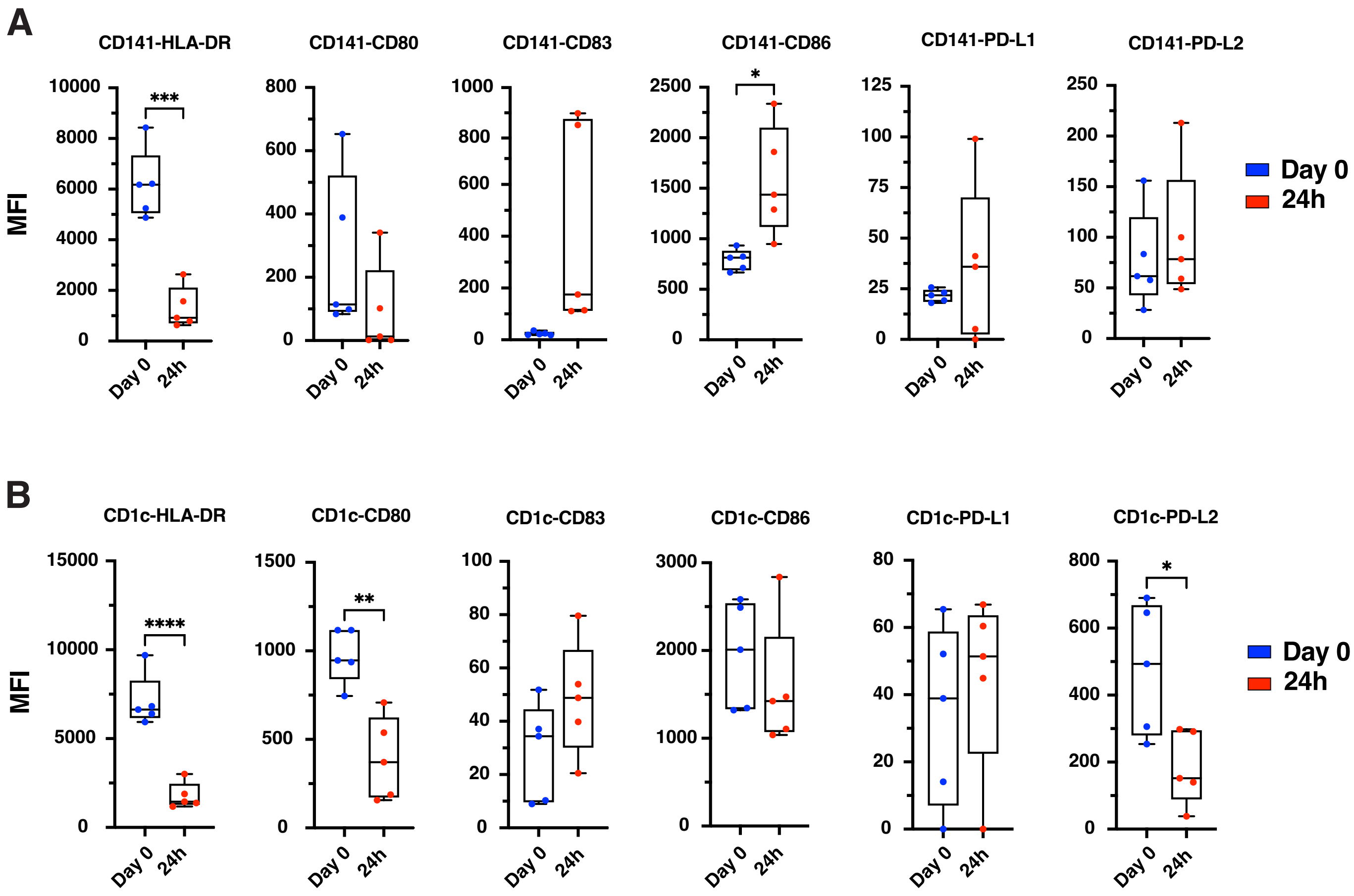

Supplementary Figure 3. The expression of phenotypic markers drops in both CD141+ and CD1c+ cells 24hrs after sorting. CD141+ and CD1c+ cells were sorted and incubated at 37 degrees in CellGenix for 24hrs. The expression of CD markers in these cells was measured right after sorting and also after 24hrs incubation. Data are presented as mean fluorescence intensity (MFI). The data is mean ± standard deviations (SD) and produced from five independent biological replicates. Student's t test analysis was used for calculation of *p* values ***, $p \leq 0.001$; ***, $p \leq 0.001$; **, $p \leq 0.01$; *, $p \leq 0.05$.
