# Supplementary material for: GM-CSF, Flt3-L and IL-4 affect viability and function of conventional dendritic cell types 1 and 2
Source: Front Immunol. 2023 Jan 12;13:1058963. doi: 10.3389/fimmu.2022.1058963 (PMC9880532; doi:10.3389/fimmu.2022.1058963)
Supplement: Supplementary file 4 [file DataSheet_4.pdf]

Supplementary Figure 4

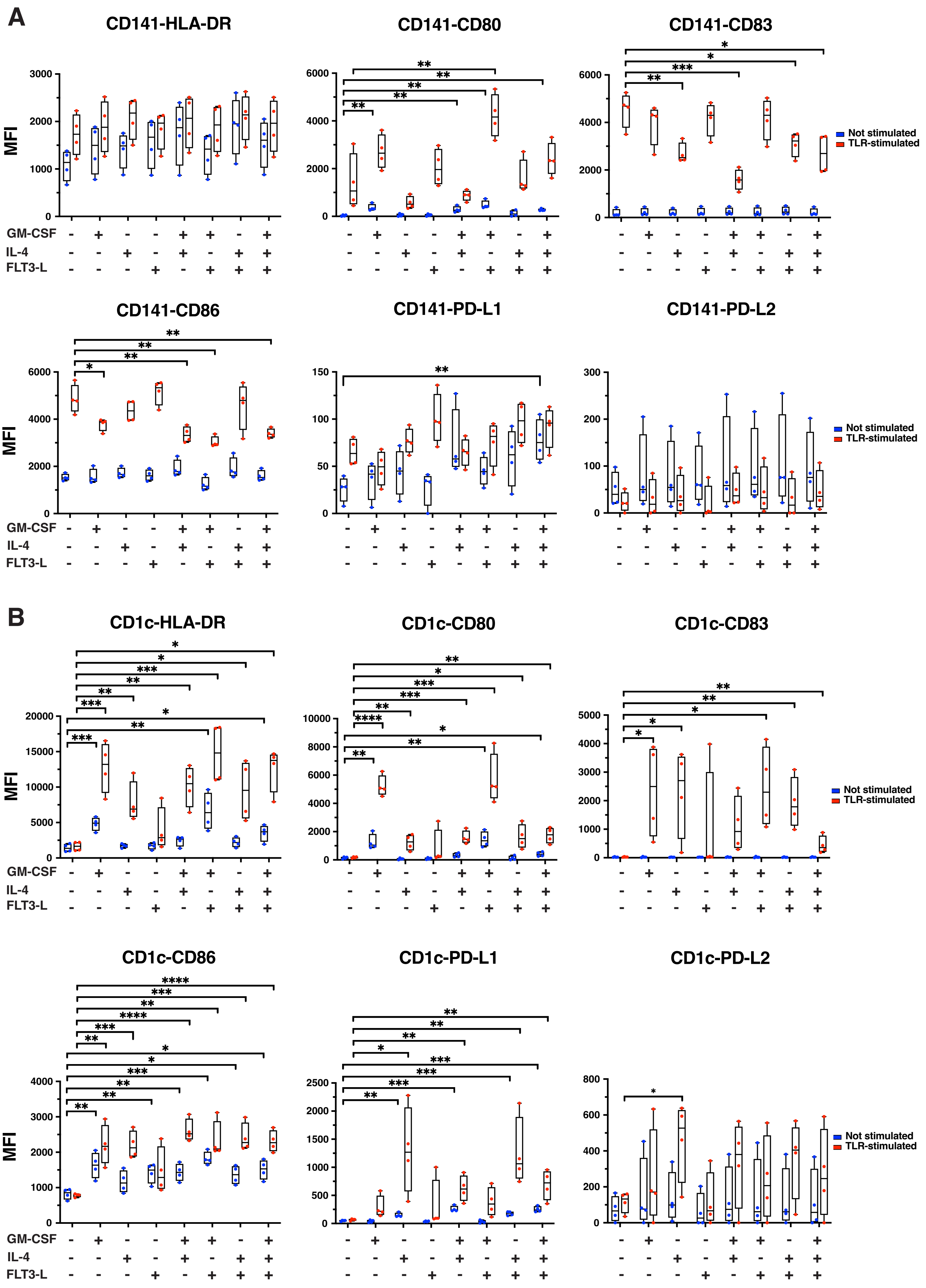

Supplementary Figure 4. The expression of CD surface markers in CD141+ and CD1c+ cells upon different cytokine treatments in the presence or absence of stimuli for 4hrs. CD141+ and CD1c+ cells were sorted and cultured in CellGenix media containing different cytokines as stated in the graph. Cells were incubated at 37degree overnight. After 20hrs (20h), cells were either treated with stimuli (CD141+ cells were treated with Poly I:C and CD1c were cells treated R848) or left untreated for 4hrs (4h). The expression of HLA-DR, CD80, CD83, CD86, PD-L1, PD-L2 were analyzed with a flow cytometer, and graphs represent mean fluorescence intensity (MFI) from 4 biological replicates. The data is mean ± standard deviations (SD). Student's *t* test analysis was used for calculation of *p* value ****, $p \leq 0.0001$; ***, $p \leq 0.001$; **, $p \leq 0.01$; *, $p \leq 0.05$; ns, not significant.
