# Supplementary material for: GM-CSF, Flt3-L and IL-4 affect viability and function of conventional dendritic cell types 1 and 2
Source: Front Immunol. 2023 Jan 12;13:1058963. doi: 10.3389/fimmu.2022.1058963 (PMC9880532; doi:10.3389/fimmu.2022.1058963)
Supplement: Supplementary file 5 [file DataSheet_5.pdf]

Supplementary Figure 5

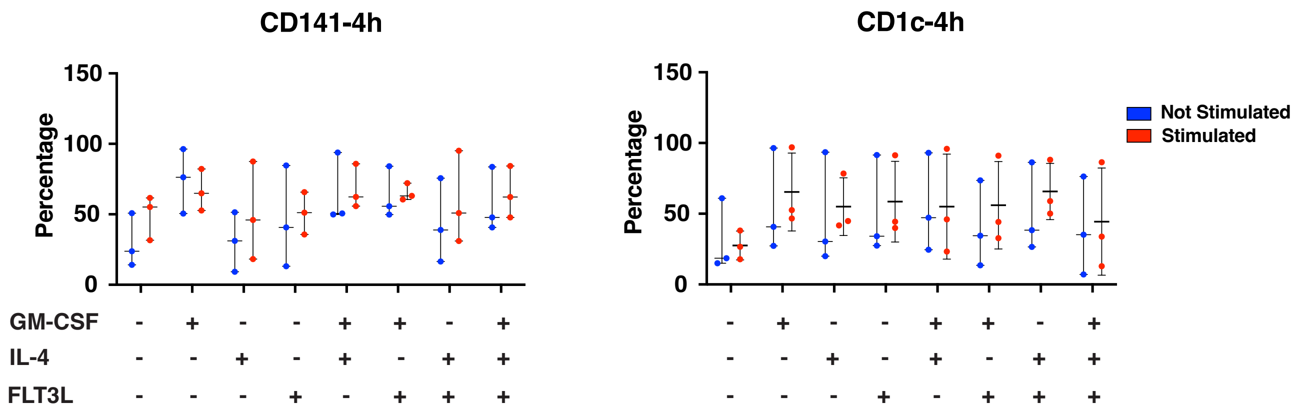

Supplementary Figure 5. Cells were prepared and as mentioned in Fig. 4B. CD141+ and CD1c+ cells were treated with stimuli only for 4h before co-culture with lymphocytes. Thereafter, cells were cultured for 8 days before flow cytometry analysis. The graphs are representative of three biological replicates.
